# Influence of acute kidney injury on high sensitive troponin after cardiac surgery. a single center retrospective observational study

**DOI:** 10.1186/2197-425X-3-S1-A633

**Published:** 2015-10-01

**Authors:** AS Omar, P Sivadasan, S Hanoura, S Sudarsanan, Y Shouman, H Ragab, AK Tuli, R Singh, A Al Khulaifi

**Affiliations:** Hamad Medical Corporation, Cardiothoracic Surgery-Heart Hospital, Doha, Qatar; Faculty of Medicine, Critical Care Medicine, Beni Suef University, Beni Suef, Egypt; Faculty of Medicine, Anaesthesiology, Alazhar University, Cairo, Egypt; Hamad Medical Corporation, Biomedical Statistics, Medical Research Center, Doha, Qatar

## Introduction

The risk assessment of cardiac troponin and other cardiac biomarkers in end-stage renal disease is not equivalent where clinical decision making in patients with renal diseases based on cardiac biomarkers needs justification in relation to patient management or outcomes [1]. Long-term outcome could be influenced by acute kidney injury (AKI) in cardiac surgery [2], but cardiac troponins need exploration in theses settings.

## Objectives

Assess the diagnostic performance of high sensitive troponin T (hsTnT) in the settings of cardiac surgery-induced AKI. Link it with mortality as well as the lengths of ventilation, ICU stay and hospital stay.

## Methods

Single center observational retrospective study. A database was available for all patients (sex, age, body mass index, duration of the operation, duration of ICU and hospital length of stay, levels of cardiac enzymes, evidence of perioperative myocardial infarction, early mortality. The lengths of ventilation, stay in ICU, and hospitalization. Based on the Acute Kidney Injury Network, AKI was defined as an abrupt (within 48 h) reduction in kidney function, defined as an absolute increase in serum creatinine concentration of 0.3 mg/dL (26.4 µmol/L) or greater or a percentage increase of 50% or greater (1.5-fold from baseline). Patients divided into 2 groups, group I without AKI (259 patients) and group II with AKI (100 patients) where serial of hsTnT and creatine kinase (CK)-MB followed. Both groups compared and statistically analyzed. We enrolled 359 patients, patients with ESRD were excluded.

## Results

**Figure 1:**
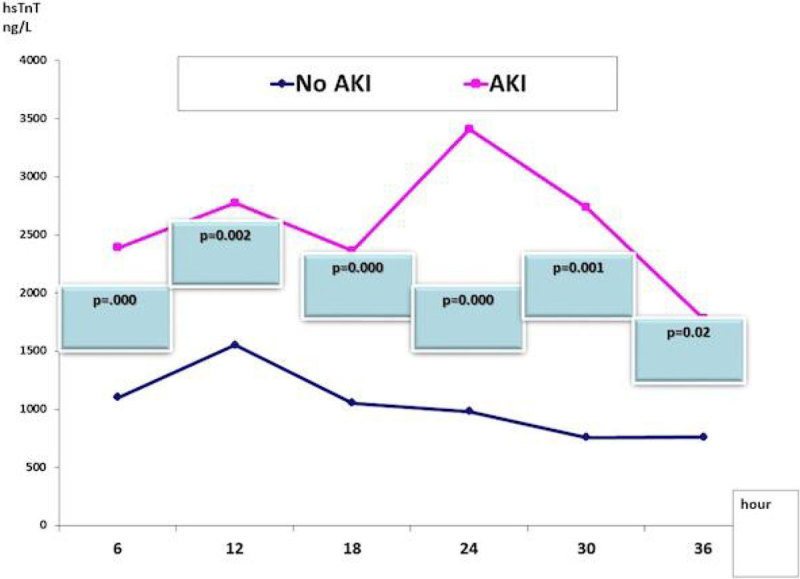
**Changes in hsTnT in both groups**

**Figure 2:**
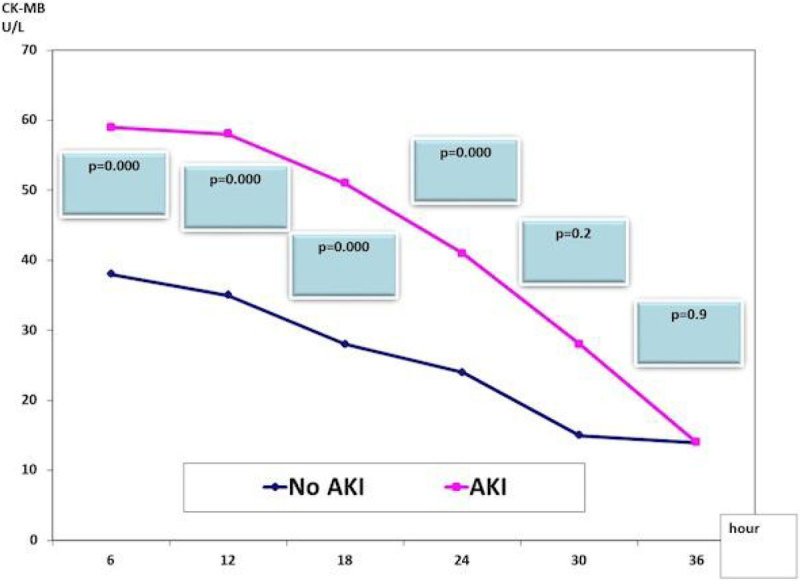
**Changes in CK-MB in both groups**

The mean age in our study population was 55.1 ± 10.2 years. High association of AKI (27.8%) was found in our patients. Both groups were matched regarding the age, gender, body mass index, the association of diabetes or hypertension, and Euro score. AKI group had significantly higher mortality 6% vs group I 1.7% (p = 0.026). The hsTnT significant changes between both groups remain all over the course, which unparalleled to those of CK-MB (Figures 1&2). The AKI group with more associated with heart failure 17.9% vs 4.9% (p=.000); and post-operative atrial fibrillation, 12.4% vs 2.9% (p = 0.005). Lengths of ventilation, stays in ICU and in hospital were significantly higher in the AKI group (Table 1).

**Table 1:** Comparison between both groups.

Variable	Group I (No AKI) 259 (%)	Group II (AKI) 100 (%)	P- Value
Age	54.43 ± 10.8	56.09 ± 10.7	0.13
Diabetes	138 (53.2)	56 (56)	0.38
Euro score	3.8 ± 2.4	5.1 ± 3.6	0.06
POAF	7 (2.7)	12 (12)	0.005
Mortality	5 (1.9)	7 (7)	0.026
LOV(minutes)	364.1 ± 112	575.5 ± 199	0.001
LOSICU (hours)	52.9 ± 41.1	109.4 ± 89	0.000
LOH (days)	10.8 ± 6.4	15.8 ± 7.3	0.007
POAF: post operative atrial fibrillation, LOSICU length of stay in ICU, LOV length of ventilation, LOH hospital length of stay			

## Conclusions

Unlike the CK-MB profile, the hsTnT showed significant changes between both groups all over the course denoting possible delayed clearance in patients with AKI that needs to put in consideration in interpreting post-operative myocardial injury and infarction in this population.

## Grant Acknowledgment

All members of CT department and medical research center, HMC, Doha, Qatar
